# In-home environment and happiness among older adults in Thailand

**DOI:** 10.3389/fpubh.2023.1159350

**Published:** 2023-04-03

**Authors:** Alongkorn Pekalee, Rossarin Soottipong Gray

**Affiliations:** ^1^Department of Health Education and Behavioral Sciences, Faculty of Public Health, Mahidol University, Bangkok, Thailand; ^2^Institute for Population and Social Research, Mahidol University, Nakhon Pathom, Thailand

**Keywords:** happiness, housing condition, in-home environment, less developed country, living condition, older adults, physical disability, structural equation modeling

## Abstract

**Background:**

The fact that housing can play a critical role in maintaining the health and independence related to happiness of the older population has been studied in more developed countries. However, research on the effect of housing conditions on happiness is rare in less developed countries. This study aimed to construct and test a structural equation model describing the structural relationship among personal aspects (living alone and physical disability), in-home environment (sleeping place and toilet/bathroom), and happiness among older adults in Thailand.

**Method:**

The data on the population age 75 years or over were extracted from the 2017 national Survey of Older Persons in Thailand (*n* = 7,829).

**Results:**

The median age of the sample population was 79. Almost 60 percent were women. The structural equation model showed a good fit with the data. Living alone did not directly influence happiness. Physical disability had a statistically significant negative direct effect on happiness. In-home environment not only had an impact on happiness directly, but also moderated the relationship between physical disability and happiness.

**Conclusion:**

The research suggested that interventions to improve happiness of older adults, particularly those with physical disability, should aim to adapt their housing, including sleeping place and toilet design.

## 1. Introduction

The factors associated with happiness among older people have been of increased interest to researchers in recent decades. This may be due not only to the accelerating pace of population ageing around the world, especially the oldest-old and increased longevity among older adults (1), but also the benefit to the nation in having a society with happy members. Longevity is, however, a pressing issue for public health, not least because of the greater likelihood of frailty and disability in older age (2).

One of the most common consequences of aging is functional health decline. The association of physical health with human well-being is well-understood. It has been found that the presence of disability is an important determinant of happiness and survival among older adults (3–7). This may lead to several studies focusing on a person-environment, fit-oriented analysis for healthy aging (a positive view of aging) (8) which is advocated by the World Health Organization’s International Classification of Functioning, Disabilities, and Health (ICF) (9). According to the ICF, statistical relationships are expected to be found between physical functioning, social participation, personal characteristics, and environmental factors, including in-home environment (10). An appropriate indoor environment (or age-friendly housing) for those with limitations includes the availability of support mechanisms (e.g., to prevent falls), having proper material for daily living, and age-friendly facility design. Health-related safety in the home needs to be addressed in happiness studies among older populations since, as people grow old, they spend more time in the home. Safety could be conceptualized as preventing or reducing the risk of problems that could undermine older people’s ability to live independently at home (11). According to the framework for health-related safety of Lau et al. (12), risk is associated with individual functioning and behavior (e.g., physical decline), and the social and physical environment (e.g., social isolation and in-home hazards), particularly among those living alone.

As population aging progresses, the prevalence of older-single-person households increases as well. There is a link between types of living arrangement (particularly living alone) and happiness among older adults. In general, those who live alone are more likely to feel lonely and less happy than those living with others. However, when an older person voluntarily chooses to have a single-person household, his/her choice may have a positive effect on their sense of well-being. It is possible that s/he may feel more stress due to various restrictions when one must live with other family members (13). Nevertheless, co-residence with kin or others is often arranged when older adults need daily personal care as they age. An inverse relationship may occur as well, i.e., very old adults living alone have a pronounced risk of losing independence and becoming socially isolated. Importantly, the in-home environment needs to be modified to support older adults living with disability or living alone in order for them to stay independently in their homes.

Appropriately modified homes may protect individuals from accidental injury, and provide them with adequate long-term housing, permitting greater autonomy and preserving social ties (14). The association between the housing environment and well-being has been studied extensively among older adults living alone in the age range of 75–89 years, especially in the European countries of Sweden, Germany, United Kingdom, Latvia, and Hungary (15, 16). For instance, Oswald, Wahl, Mollenkopf, and Schilling (17) conclude that housing conditions played an important role in life satisfaction for older people (age 55–99 years) in two rural regions of Germany. Similarly, quality housing and a feeling of home attachment were associated with psychological well-being among the population age 60 years or over living independently (alone or with another older adult) in the United States (18). In urban China, a study found that housing conditions, housing satisfaction, and home ownership had an impact upon life satisfaction among those between 18 and 69 years of age (19). Also, in China, a recent study found that the housing environment was associated with depressive symptoms among older adults aged 60 or over (20). Nevertheless, studies that have examined the relationship between housing conditions and happiness among older adults are rare in less-developed countries.

Thailand is a middle-income country in Asia where aging is occurring very rapidly, and Thailand is currently ranked 8th in Asia in terms of the percentage of the population age 60 years or over. In 2019, these older adults accounted for 18 percent of the total Thai population, and the proportion is projected to reach 28 percent by the year 2037. The rate of population growth is highest among the “oldest-old” (i.e., 80 years or over) (21). Many of these older persons may have difficulty in performing activities in daily living (ADL), and the majority will spend most of their time in their home residence. The challenge is how to enable older people in Thailand, especially those in the mid-and oldest-old groups who had high dead rate from fall (22), to live safely and happily in their home environment, given the traditional Thai filial piety norms and government policy of “aging in place” (23).

Apart from rapid population aging, Thailand has undergone major socioeconomic and cultural changes in recent decades. The composition of Thai households has become more diverse and characterized by an increased prevalence of persons aged 60 years or over living alone: from 6 percent in 2002 to 11 percent in 2017 (21). Many Thai people have adopted a number of westernized, health-promoting lifestyles, e.g., switching from sleeping on the floor to sleeping on a bed, or using a sit-down toilet instead of a squat latrine (24). Previous studies of happiness among older Thais found that economic hardship, relative poverty, living arrangement, functional ability, social environment, family and friendship support, and healthy lifestyle behaviors were associated with happiness, psychological well-being, and/or life satisfaction (25–27). No study, however, includes the in-home environment as a determinant of happiness of older persons in Thailand. In addition, most studies in and outside of Thailand are cross-sectional, and use non-causal relationship analyses. Thus, this study attempted to fill gaps in the national and international research literature and test an analytical model of the structural relationship among personal aspects, in-home environment aspects, and happiness among older adults in Thailand (controlling for other generally acknowledged determinants). This report concludes with suggested interventions, based on causal-relationship findings, to support Thailand’s “aging-in-place” policy.

## 2. Materials and methods

### 2.1. Research model and hypothesis setting

**Figure 1:**
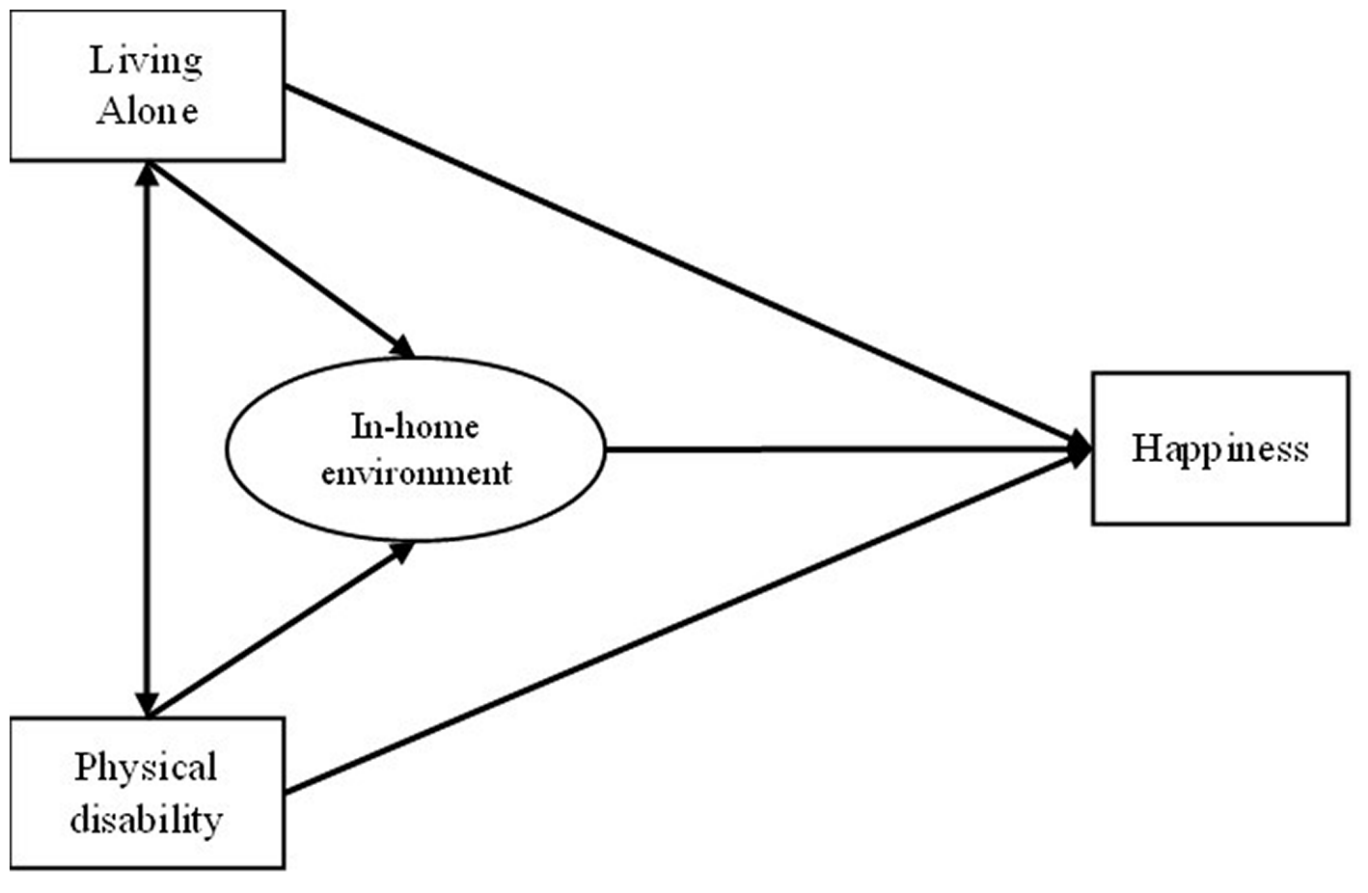
Hypothesized model.

Based on the literature review, a causal model of the relationship between personal factors, in-home environment, and happiness is proposed (Figure 1). The study hypothesis assumes two exogenous variables, one mediating variable and one endogenous variable. The two exogenous variables are living alone and physical disability. Happiness is the endogenous variable. In-home environment is considered a mediating variable that influences the relationship between the two exogenous variables and happiness. Thus, the present study analyzed the direct, indirect, and total effects of exogenous variables, and the direct effects of the mediating variable on happiness.

### 2.2. Data collection and procedures

Data for this study were derived from the Survey of Older Persons in Thailand, which was carried out by the Thai National Statistical Office (NSO) in 2017. That survey includes a representative sample of 41,752 persons aged 60 years or over. The NSO used a two-stage stratified sampling design. The first stage included sample blocks in municipal areas and sample villages in rural areas in all provinces of Thailand. Private households were sampled in the second stage, and those age 60 years or older in all selected households were interviewed face-to-face. However, for 8,995 cases, information was obtained by proxy response (i.e., from members in the household and/or non-household members), and these cases were excluded from the analysis in the present study. Thus, there were 32,757 respondents interviewed directly. Persons aged 75 years or older were selected for this study since loss of independence rapidly increases with advanced age in most individuals. Thus, the final sample comprises 7,829 respondents. The results were weighted, and Structural Equation Modeling (SEM) was used to test the hypotheses.

SEM is a statistical model which displays the causal relationships among several variables in a path diagram (28). SEM is also referred to as a combination of multiple regression and factor analysis. SEM is used to reduce the limitation of regression equations that allow the occurrence of an error of measurement in the endogenous and exogenous variables. In addition, SEM allows interpretation of the direct (linear influences) and indirect effects (nonlinear influences) among the study variables (29). SEM was performed using the software package AMOS Version 18. This study was approved by the Institutional Review Board (IRB) of the Institute for Population and Social Research of Mahidol University (COE. No. 2021/06–122).

### 2.3. Assessment of study variables

The respondents were asked to assess their level of happiness based on the following question: “In the past 3 months, what was the level of your happiness?” The potential response scores range from 0 to 10, where 0 means “unhappiest” and 10 “happiest.” A single-item instrument referring to happiness is commonly used for well-being research (30, 31). One study found that measuring happiness using a single question was reliable and valid since the answers had a high positive correlation with those provided by other happiness scales or inventories (32).

A favorable in-home environment for independent living in this study refers to a situation in which “The physical surroundings in the house support older occupants to perform ADL, including having a bed, having a sit-down toilet, presence of handrails in the bedroom/toilet/bathroom, and having an in-house toilet.” In-home environment was assessed using four items: Sleeping place, toilet/bathroom, presence of handholds in both these settings, and location of the toilet -- indoors or outdoors. The first question was as follows: “Where do you sleep?” with potential answers of on the floor or on a bed. The second question was: “What type of toilet do you use?” with potential answers of sit-down toilet or squat latrine. The third question was “Does your house have handrails in the bedroom/toilet/bathroom?” with potential answers yes or no. The fourth question was “Where is your toilet/bathroom located?” with potential answers of in the house or outside the house. Confirmatory Factor Analysis was used to test whether these components can be appropriately aggregated into a single in-home environment construct. It was found that the five components of the in-home environment model indicated overall good fit according to various fit indices: *χ*^2^ = 86.626 (*p* < 0.001), GFI = 0.953, NFI = 0.953, CFI = 0.953, and RMSEA = 0.05, indicating the model had good fit (28, 33).

Physical disability was assessed according to the following eight ADL: bathing, dressing, toileting, continence, feeding, getting up from a lying down position, squatting, and climbing 2–3 stairs. The question was “Can you perform (the above ADL) … by yourself?” The potential answers were classified into two groups: Yes, could do by self (score = 0), and with difficulty (i.e., could not do at all or need assistance) (Score = 1). The possible score range is 0 to 8, with the lowest score denoting no physical disability, and the highest score denoting dependence on others for all ADL.

Living arrangement was categorized into “living alone” or “living with other (s).” It was based on the question “How many persons live in this household?” In this study, living alone refers to older adults living in this household without anyone else.

Demographic and socioeconomic characteristics including age, sex, marital status, educational attainment, and personal income were also included in the analysis as control variables. These factors were found to be associated with happiness among older adults (5).

## 3. Results

### 3.1. Characteristics of the sample

Table 1 shows that more than half the total sample were those age 75–79 years while persons aged 80 years or over constituted 45.8 percent. Almost 60 percent were females, and 45.6 percent of the sample were currently married. About three-fourths had completed only primary school, and half the sample had personal income under 30,000 baht (about $1,000) per year.

**Table 1:** Characteristics of the sample and mean happiness score (*n* = 7,829).

Characteristics	%	Happiness Score	
		Mean	SD
*Age group* (Mean = 80.08, Median = 79.00, SD = 4.39, Min = 75 Max = 103)			
75–79	54.2	6.86	1.40
80 or over	45.8	6.70	1.43
*Sex*			
Male	40.7	6.82	1.40
Female	59.3	6.77	1.43
*Marital status*			
Single, Widowed, Divorced	54.4	6.77	1.42
Married	45.6	6.81	1.42
*Educational attainment*			
No formal education	14.2	6.60	1.45
Primary school	77.4	6.76	1.40
Secondary school	6.0	7.26	1.39
Bachelor’s or higher degree	2.4	7.64	1.41
*Personal income per year* (*baht*)			
Less than 10,000	15.2	6.50	1.48
10,000–29,999	38.5	6.54	1.39
30,000 - 49,999	21.3	6.95	1.35
50,000 - 79,999	14.3	7.03	1.33
80,000 or above	10.7	7.45	1.37
*Living arrangement*			
Alone	16.7	6.71	1.45
With other(s)	83.3	6.80	1.41
*Physical disability*			
0	60.3	6.94	1.34
1	21.4	6.77	1.43
2	12.1	6.47	1.50
3	3.3	6.11	1.43
4	0.9	6.07	1.44
5	0.6	5.80	1.75
6	0.5	5.59	1.67
7	0.5	5.08	1.86
8	0.4	5.56	1.54
*Sleeping place*			
Floor	40.5	6.59	1.40
Bed	59.5	6.92	1.42
*Handrails in bedroom*			
No	39.0	6.66	1.46
Yes	61.0	6.87	1.39
*Toilet type*			
Squat latrine	48.3	6.57	1.38
Sit-down	51.7	6.99	1.42
*Handrails in toilet/bathroom*			
No	86.9	6.76	1.42
Yes	13.1	6.99	1.40
*Location of toilet/bathroom*			
Outside the house	18.4	6.48	1.41
Inside the house	81.6	6.86	1.41

About one in six of these older Thais lived alone (16.7 percent). Three out of five had no physical disability, while about one in five had difficulty in performing one ADL. Regarding in-home environment, three out of five respondents slept on a bed while the rest slept on a mat or mattress on the floor. Almost the same proportion (61 percent) had handrails in the bedroom. About half the sample used a sit-down toilet, but only 13 percent had handrails next to the toilet. Four out of five said that their toilet was located inside the house.

### 3.2. SEM analysis

**Figure 2:**
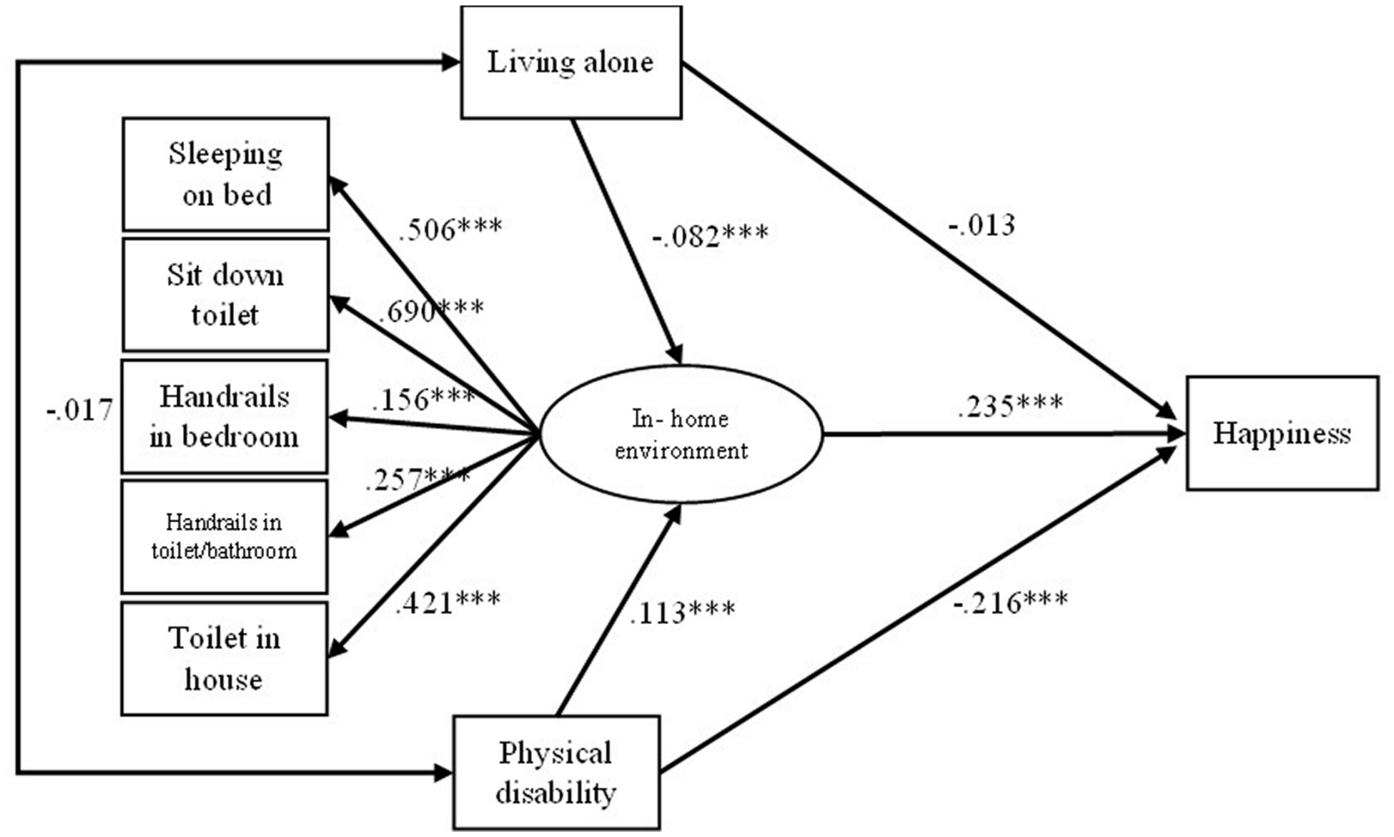
Structural model of happiness of Thai older adults. Control variables were age, sex, marital status, educational attainment, and personal income per year; ^*^p < 0.05, ^**^p < 0.01, ^***^p < 0.001; Model fit indices: *χ*^2^ = 302.397 (*p* < 0.001), GFI = 0.964, NFI = 0.959, CFI = 0.964, and RMSEA = 0.10.

The SEM model and its standardized direct and indirect coefficients (controlled for age, sex, marital status, educational attainment, and personal income) are presented in Figure 2. The coefficients for direct, indirect, and total effects with confidence intervals are described in Tables 2, 3.

**Table 2:** Direct effects of the model controlling for age, sex, marital status, educational attainment, and personal income per year (standardized regression coefficients).

Direct effects	Factor loadings (β) (95% CI)
Physical disability ↔ Living alone	−0.017 (−0.038 to 0.003)
Living alone → In-home environment	−0.082^***^ (−0.113 to −0.054)
Physical disability → In-home environment	0.113^**^ (0.086 to 0.140)
Age → In-home environment	0.046^**^ (0.018 to 0.073)
Gender → In-home environment	−0.100^***^ (−0.130 to −0.070)
Marital status → In-home environment	0.038^*^ (0.005 to 0.070)
Education → In-home environment	0.334^***^ (0.310 to 0.358)
Personal income → In-home environment	−0.154^***^ (−0.181 to −0.126)
In-home environment → Happiness	0.235^***^ (0.203 to 0.266)
Living alone → Happiness	−0.013 (−0.038 to 0.011)
Physical disability → Happiness	−0.216^***^ (−0.241 to −0.193)
Age → Happiness	−0.026^*^ (−0.049 to −0.004)
Gender → Happiness	0.011 (−0.013 to 0.035)
Marital status → Happiness	−0.034^**^ (−0.060 to −0.009)
Education → Happiness	0.030^*^ (0.004 to 0.057)
Personal income → Happiness	−0.033^**^ (−0.055 to −0.010)

^*^p < 0.05, ^**^p < 0.01, ^***^p < 0.001.

**Table 3:** Indirect and total effects of the research model controlling for age, sex, marital status, educational attainment, and personal income per year.

Path diagram	Factor loadings (β) (95% CI)		
	Direct effects	Indirect effects	Total effects
Living alone → In-home environment → Happiness	−0.013 (−0.038 to 0.011)	−0.019^***^ (−0.028 to −0.013)	−0.032^**^ (−0.056 to −0.009)
Living alone → Physical disability → Happiness		0.000 (0.000 to 0.000)	
Living alone → Physical disability → In-home environment → Happiness		0.000 (0.000 to 0.000)	
Physical disability → In-home environment → Happiness	−0.216^***^ (−0.241 to −0.193)	0.026^***^ (0.019 to 0.034)	−0.190^***^ (−0.216 to −0.166)
Physical disability → Living alone → Happiness		0.000 (0.000 to 0.000)	
Physical disability → Living alone → In-home environment → Happiness		0.000 (0.000 to 0.000)	

^*^p < 0.05, ^**^p < 0.01, ^***^p < 0.001.

The SEM model showed a good fit with the data, *χ*^2^ = 302.397 (*p* < 0.001), goodness of fit index (GFI) = 0.964, normed fit index (NFI) = 0.959, comparative fit index (CFI) = 0.964, and root mean square error of approximation (RMSEA) = 0.10. Among the direct effects, the analysis found that in-home environment was the most significant predictor of happiness among Thai older adults (0.235). Physical disability had a significantly negative effect on happiness (−0.216) and a significantly positive effect on in-home environment (0.113). Living alone did not significantly affect happiness (−0.013), but it had a significantly negative impact on in-home environment (−0.082). This study also found that living alone was not significantly predicted by physical disability (−0.017) and vice versa.

In-home environment was defined as a mediating variable in the research model to verify mediation effect significance among exogenous variables (i.e., living alone and physical disability) and the endogenous variable (happiness). The analysis found a significant indirect effect of living alone and physical disability on happiness (−0.019 and 0.026 respectively) (Table 3). It should also be noted that the indirect effect of living alone on happiness, as mediated by physical disability alone and physical disability plus in-home environment, was not significant and equal to 0.000, suggesting that there could be no effect. Similarly, the indirect effect of physical disability on happiness mediated by living alone and living alone plus in-home environment was not significant and equal to 0.000, suggesting that there could be no effect as well.

The total effects (shown in Table 3) indicate the greater importance of physical disability on happiness than living alone. Moreover, the negative coefficient of the total effect of physical disability decreased compared with the direct effect (from −0.216 to −0.190), suggesting that the mediating factor (in-home environment) helped increase happiness among older adults with physical disability.

## 4. Discussion

The present study investigated whether the role of the in-home environment had a significant impact on happiness directly and/or moderated by the relationship between living independence (i.e., living alone and physical disability) in a national sample of Thais age 75 years or over. The SEM model presented a good fit, indicating that our hypotheses offered a plausible explanation of how the in-home environment is related to happiness, when controlling for the potential confounding effect of age, sex, marital status, educational attainment, and personal income.

### 4.1. Direct effect

Among the direct effects, happiness was mainly predicted by in-home environment (i.e., sleeping place and type of toilet): Older adults living in a better in-home environment were happier. This finding is similar to that of previous studies (18). However, living alone had no significant effect on happiness. That finding is inconsistent with previous studies. For example, a study by Hwang and Sim (31) found significant differences in happiness between types of living arrangement in which those living alone had the least happiness.

Those who had a higher level of disability were less happy, and that finding is consistent with many previous studies (4, 5). The significant negative direct link between living alone and in-home environment suggests that older adults who live alone were less likely to live in a better in-home environment. Those living alone tend to be a population subgroup selected for those with good health. Thus, they do not need in-home facilities to support ADL. This finding is consistent with the result of the analysis which found that living alone was not predicted by physical disability, and vice versa. This may be because most respondents in the sample had no disability or only a single physical disability (Table 1). As expected, those with physical disability were more likely to live in a better in-home environment, i.e., to support them in ADL.

### 4.2. Mediating effect

The analysis found that the path from living alone and physical disability to happiness is mediated by in-home environment. The path from living alone → in-home environment → happiness was significant with a negative coefficient. The total effects of living alone on happiness had a significantly negative increased coefficient compared to its direct and indirect effects. These findings suggest that, although older adults in single-person households lived in a better in-home environment, they may prefer living with others (i.e., family members). This is likely to be the case in many Asian countries such as China (13) and Thailand (34, 35), particularly when persons reached advanced age and need daily personal care. The significant, indirectly positive effect of physical disability → in-home environment → happiness, and the significant total effect of physical disability → happiness demonstrates that a more comfortable in-home environment (i.e., sleeping place and type of toilet) helped increase a sense of happiness among older adults with physical disability.

These findings support the Thai government policy of “*aging in place*” for the rapidly growing population of older adults (particularly the oldest-old) by identifying factors that predict self-perceived happiness persons aged 75 years or over and indicating which kinds of individuals can benefit from the modification of in-home environment (i.e., frailty in performing ADL). Two dimensions of the in-home environment (sleeping place and toilet/bathroom) were assessed in this study. The bathroom (i.e., availability of handrails, and toilet type) is a key factor since it is the most unsafe room in an older adult’s home (36).

The findings reveal that the in-home environment of older Thais needs to be improved. About two in five older persons slept on the floor, and about the same proportion had no handrails in the bedroom. Although about four out of five older Thais had an indoor toilet/bathroom, about half used a squat latrine, and only one in ten had handrails in their toilet/bathroom. Housing without the proper sleeping place, elder-friendly toilet, and amenities to support frail older persons could increase the rate of accidental falls and injury among this growing segment of the population. Similar housing conditions were documented in another study in Thailand (37), and that study found that many older people slept on a thin mat or mattress on the floor.

Older adults who slept on a bed, used a sit-down toilet, had handrails in the toilet/bathroom, and had an indoor toilet were happier than their counterparts who slept on the floor, used squat latrine, had no handrails in the toilet/bathroom, and had an outdoor toilet. Those with limited function ability may find it challenging to lie down on the floor and get back up again multiple times during the night. The presence of handrails in the toilet/bathroom and having an indoor toilet helped older persons in carrying out ADL. Additionally, the older adults (particularly those with physical disability) did not have to worry about hazards in the course of performing everyday functions.

That happiness among older adults with physical disability was mediated by in-home environment can be explained by the ecology theory of aging (ETA) (38). According to ETA, individuals with low functional capacity are much more vulnerable to environmental demands than those with high capacity. In addition, aspects of the living environment (e.g., sleeping place, type/location of toilet) are critical to what older persons can manage in their everyday lives. Thus, those who lived in a poorer housing environment felt less happy. However, the present study suggests that it should be possible to increase happiness of older persons by modifying the structure and amenities of the household. Thailand has been recognized as a success story for converting the population from defecating in open spaces or into a squat pit privy to using a sanitary latrine. A nationwide health education campaign was waged over many years to convince the population of how a sanitary latrine was healthier and a way to eliminate foul odors. Currently, the Thai Ministry of Public Health has a policy to replace all the squat latrines around the country with sit-down toilets. This policy was formulated in recognition of the exploding population of older persons and to reduce discrimination against persons with disabilities (24). That said, Thai families have limited knowledge about how to adapt their home to be elder-friendly. Fortunately, senior-friendly accommodations are increasingly used as a marketing tool in the Thai real estate sector, and the government plans to provide more of this type of affordable housing for middle-and lower-income older persons. In addition, a government allowance of up to 100,000 baht per household (~ $3,000) is available to help communities renovate the homes of older persons in order to make them safe and suitable for aging bodies (39, 40). It should also be noted that a Mexican government program to replace dirt floors with cement significantly improved the health of young children, as measured by decreases in the incidence of parasitic infestations and diarrhea, a decrease in the prevalence of anemia, and an improvement in childhood cognitive development. Additionally, the Mexican program demonstrated significant improvements in adult welfare as measured by increased satisfaction with housing and quality of life, as well as by lower scores on depression and self-perceived stress scales (41).

### 4.3. Limitation of this study

Because the data set was secondary, only a limited number of housing characteristics for ADL were available (i.e., sleeping place, type of toilet, handrails, location of toilet). Nevertheless, despite these limitations, the analysis was able to identify key in-home design variables that contribute to older adult-friendly housing. Future studies should include more refined indicators of housing quality (e.g., kitchen area, floor material, etc.,).

In addition, cross-national interpretation of housing-related findings should be cautious. The findings from this study may be applicable to less-developed countries for two reasons. First, the measurement of suitable in-home environment for older adults are likely to be different between less developed and developed countries depending on differences in housing standard (15). Secondly, the living arrangements of older adults and, more specifically, living alone are the result of prevailing of cultural norms, the preferences and the resources people have, and the constraints they face as they age, such as the support from their families and public welfare. The prevalence of living alone is considerably higher in more developed countries (42). These amenities are widely different by level of country development.

## 5. Conclusion

This study found that the in-home environment of Thais age 75 years or over needs to be improved (e.g., sleeping place and toilet/bathroom). Sleeping on a bed, using a sit-down toilet, having handrails in both places, and having an indoor toilet had a positive, statistically significant direct effect on happiness of this sample of older persons. Physical disability also had a statistically significant negative direct effect on happiness. Additionally, in-home environment not only has an impact on happiness directly, but also moderates the relationship between physical disability and happiness. Therefore, there is a strong need for programs to ensure a safe living environment (e.g., adapted housing appropriate for ADL) for older adults in general, and for those with physical disability in particular.

## Data availability statement

The original contributions presented in the study are included in the article/supplementary material, further inquiries can be directed to the corresponding author.

## Ethics statement

The studies involving human participants were reviewed and approved by Institutional Review Board (IRB) of the Institute for Population and Social Research of Mahidol University (COE. No. 2021/06-122). Written informed consent for participation was not required for this study in accordance with the national legislation and the institutional requirements.

## Author contributions

RG was responsible for the conception and design of this study. AP was responsible for data analysis. AP and RG were responsible for drafting and revising the manuscript and final approval of the manuscript submitted. All authors contributed to the article and approved the submitted version.

## Funding

This work was financially supported by the International Life Sciences Institute (ILSI) Southeast Asia Region. The funder had no role in study design, data collection, data analysis, decision to publish, or preparation of the manuscript.

## Conflict of interest

The authors declare that the research was conducted in the absence of any commercial or financial relationships that could be construed as a potential conflict of interest.

## Publisher’s note

All claims expressed in this article are solely those of the authors and do not necessarily represent those of their affiliated organizations, or those of the publisher, the editors and the reviewers. Any product that may be evaluated in this article, or claim that may be made by its manufacturer, is not guaranteed or endorsed by the publisher.

## References

[1] United Nations, Department of Economic and Social Affairs Population Division. (2019). World population prospects: The 2019 revision.

[2] Kane R. Making aging a public health priority. J Am Public Health Assoc. (1994) 84:1213–4. doi: 10.2105/ajph.84.8.1213, PMID: 8059873 PMC1615463

[3] Jakobsson U Hallberg IR Westergren A. Overall and health related quality of life among the oldest old in pain. Qual Life Res. (2004) 13:125–36. doi: 10.1023/B:QURE.0000015286.68287.66 15058794

[4] Van Campen C Iedema J. Are persons with physical disabilities who participate in society healthier and happier? Structural equation modelling of objective participation and subjective well-being. Qual Life Res. (2007) 16:635–45. doi: 10.1007/s11136-006-9147-3, PMID: 17268932 PMC2798044

[5] George LK. Still happy after all these years: research frontiers on subjective well-being in later life. J Gerontol B Psychol Sci Soc Sci. (2010) 65B:331–9. doi: 10.1093/geronb/gbq006, PMID: 20233742

[6] Angner E Ghandhi J Purvis KW Amante D Allison J. Daily functioning, health status, and happiness in older adults. J Happiness Stud. (2013) 14:1563–74. doi: 10.1300/j083v38n03_02

[7] Chei CL Lee JM Ma S Malhotra R. Happy older people live longer. Age Ageing. (2018) 47:860–6. doi: 10.1093/ageing/afy128, PMID: 30165421

[8] Fernández-Ballesteros R Robine JM Walker A Kalache A. Active aging: a global goal. Curr Gerontol Geriatr Res. (2013) 2018:298012. doi: 10.1155/2013/298012, PMID: 23476642 PMC3586450

[9] Iwarsson S Wahl HW Nygren C Oswald F Sixsmith A Sixsmith J. Importance of the home environment for healthy aging: conceptual and methodological background of the European ENABLE-AGE project. Gerontologist. (2007) 47:78–84. doi: 10.1093/geront/47.1.78, PMID: 17327543

[10] World Health Organization. International classification of functioning, disability, and health (ICF). Geneva: World Health Organization (2001).

[11] Lette M Ambugo EA Hagen TP Nijpels G Baan CA De Bruin SR. Addressing safety risks in integrated care programs for older people living at home: a scoping review. BMC Geriatr. (2020) 20:81–13. doi: 10.1186/s12877-020-1482-7, PMID: 32111170 PMC7048120

[12] Lau DT Scandrett KG Jarzebowski M Holman K Emanuel L. Health-related safety: a framework to address barriers to aging in place. Gerontologist. (2007) 47:830–7. doi: 10.1093/geront/47.6.830, PMID: 18192636

[13] Matsuura T Ma X. Living arrangements and subjective well-being of the elderly in China and Japan. J Happiness Stud. (2021):1–46. doi: 10.1007/s10902021004300

[14] Gitlin LN. Conducting research on home environments: lessons learned and new directions. Gerontologist. (2003) 43:628–37. doi: 10.1093/geront/43.5.628, PMID: 14570959

[15] Iwarsson S Wahl HW Nygren C. Challenges of cross-national housing research with older persons: lessons from the ENABLE-AGE project. Eur J Ageing. (2004) 1:79–88. doi: 10.1007/s10433-004-0010-5, PMID: 28794705 PMC5547685

[16] Granbom M Iwarsson S Kylberg M Pettersson C Slaug B. A public health perspective to environmental barriers and accessibility problems for senior citizens living in ordinary housing. BMC Public Health. (2016) 16:772. doi: 10.1186/s12889-016-3369-2, PMID: 27514631 PMC4982418

[17] Oswald F Wahl HW Mollenkopf H Schilling O. Housing and life satisfaction of older adults in two rural regions in Germany. Res Aging. (2003) 25:122–43. doi: 10.1177/0164027502250016

[18] Evans GW Kantrowitz E Eshelman P. Housing quality and psychological well-being among the elderly population. J Gerontol B Psychol Sci Soc Sci. (2002) 57:P381–3. doi: 10.1093/geronb/57.4.p381 12084789

[19] Zhang F Zhang C Hudson J. Housing conditions and life satisfaction in urban China. Cities. (2018) 81:35–44. doi: 10.1016/j.cities.2018.03.012

[20] Chen Y Cui PY Pan YY Li YX Waili N Li Y. Association between housing environment and depressive symptoms among older people: a multidimensional assessment. BMC Geriatr. (2021) 21:259–10. doi: 10.1186/s12877-021-02207-9, PMID: 33865321 PMC8052816

[21] Institute for Population and Social Research, Mahidol University, Foundation of Thai Gerontology Research and Development Institute. Situation of the Thai elderly 2019. Nakhon Pathom: Institute for Population and Social Research, Mahidol University (2019).

[22] Sri-Chang N Khawee L. Prediction of Fall Among the Elderly (Age 60 Years or Over) in Thailand, 2017–2021 Division of Non-communicable diseases Ministry of Public Health (2017).

[23] Department of Older Persons. Thailand Implementation of the Madrid International Plan of Action on Ageing (Mipaa), 2002–2016. Bangkok: Amarin Printing & Publishing Public Company Limited (2016).

[24] Shirai Y Podhisita C Tipsuk P. Latrine development in Thailand. Sanitation Value Chain. (2020) 4:21–36. doi: 10.34416/svc.00024

[25] Gray RS Rukumnuaykit P Kittisuksathit S Thongthai V. Inner happiness among Thai elderly. J Cross-Cult Gerontol. (2008) 23:211–24. doi: 10.1007/s10823-008-9065-7, PMID: 18389354

[26] Thanakwang K Ingersoll-Dayton B Soonthorndhada K. The relationships among family, friends, and psychological well-being for Thai elderly. Aging Ment Health. (2012) 16:993–1003. doi: 10.1080/13607863.2012.692762, PMID: 22681447

[27] Phulkerd S Thapsuwan S Chamratrithirong A Gray RS. Influence of healthy lifestyle behaviors on life satisfaction in the aging population of Thailand: a national population-based survey. BMC Public Health. (2021) 21:43–10. doi: 10.1186/s12889-020-10032-9, PMID: 33407252 PMC7789197

[28] Hair JF Babin BJ. Multivariate Data Analysis. India: Cengage (2018).

[29] Bollen KA Long JS. Tests for structural equation models: introduction. Sociol Methods Res. (1992) 21:123–31. doi: 10.1177/0049124192021002001

[30] Sohn K. The fatter are happier in Indonesia. Qual Life Res. (2017) 26:393–402. doi: 10.1007/s11136-016-1403-6, PMID: 27582170

[31] Hwang EJ Sim IO. Association of living arrangements with happiness attributes among older adults. BMC Geriatr. (2021) 21:100–14. doi: 10.1186/s12877-021-02017-z, PMID: 33541268 PMC7860621

[32] Abdel-Khalek AM. Measuring happiness with a single-item scale. Soc Behav Pers. (2006) 34:139–50. doi: 10.2224/sbp.2006.34.2.139

[33] Schermelleh-Engel K Moosbrugger H Müller H. Evaluating the fit of structural equation models: tests of significance and descriptive goodness-of-fit measures. Methods Psychol Res Online. (2003) 8:23–74.

[34] Thongsrikate I. The happiness of elderly migrants who moved to the City of Khon Kaen, Khon Kaen Province. Suranaree J Soc Sci. (2020) 14:73–88.

[35] Wongsala M Anbäcken EM Rosendahl S. Active ageing–perspectives on health, participation, and security among older adults in northeastern Thailand–a qualitative study. BMC Geriatr. (2021) 21:1–10. doi: 10.1186/s12877-020-01981-2, PMID: 33430777 PMC7802255

[36] Feldman F Chaudhury H. Falls and the physical environment: a review and a new multifactorial falls-risk conceptual framework. Can J Occup Ther. (2008) 75:82–95. doi: 10.1177/000841740807500204 18510252

[37] Jarutach T Lertpradit N. Housing conditions and improvement guidelines for the elderly living in urban areas: case studies of four Bangkok’s districts. N J Environ Design Plann. (2020) 18:117–38. doi: 10.54028/NJ202018117138

[38] Lawton M. P. (1999). Environmental taxonomy: Generalizations from research with older adults.

[39] Asian Development Bank. (2021), Country Diagnostic Study on Long-Term Care in Thailand. 2020. Available at: https://www.adb.org/sites/default/files/publication/661736/thailand-country-diagnostic-study-long-term-care.pdf

[40] Department of Older Persons. (2021). Handbook for Adapted Home for Safety for 2020 Financial Year 2020. Available at: https://www.dop.go.th/download/formdownload/th1593742686-832_0.pdf

[41] Cattaneo MD Galiani S Gertler PJ Martinez S Titiunik R. Housing, health, and happiness. Am Econ J Econ Policy. (2009) 1:75–105. doi: 10.1257/pol.1.1.75

[42] Reher D Requena M. Living alone in later life: a global perspective. Popul Dev Rev. (2018) 44:427–54. doi: 10.1111/padr.12149

